# Resection of pancreatic and splenic metastases from alveolar soft part sarcoma: a case report

**DOI:** 10.1186/s40792-020-00907-9

**Published:** 2020-06-17

**Authors:** Satoshi Arakawa, Yukio Asano, Norihiko Kawabe, Hidetoshi Nagata, Yuka Kondo, Shinpei Furuta, Masahiro Shimura, Chihiro Hayashi, Takayuki Ochi, Kenshiro Kamio, Toki Kawai, Hironobu Yasuoka, Takahiko Higashiguchi, Shin Ishihara, Masahiro Ito, Yoshihiro Imaeda, Akihiko Horiguchi

**Affiliations:** Department of Gastroenterological Surgery, Fujita Health University Bantane Hospital, 3-6-10 Otobashi, Nakagawa-ku, Nagoya, Aichi 454-8509 Japan

**Keywords:** Metastases, Pancreatic tumor, Metastasectomy, Alveolar soft part sarcoma

## Abstract

**Background:**

We present a case of pancreatic and splenic metastases following alveolar soft part sarcoma (ASPS), which was successfully treated by surgery.

**Case presentation:**

A 41-year-old male was referred to our hospital in 2012. Computed tomography (CT) showed the presence of a pancreatic tumor. In 2002, the patient had undergone surgical resection of an ASPS of the anal region. In 2009, during follow-up, CT revealed lung metastases, which prompted surgical resection of the lung, followed by resection of the head skin in 2011. Abdominal ultrasonography (US) revealed the presence of isodense masses sized 34 mm in the pancreatic head and 60 mm within the spleen. The contrast-enhanced US revealed a solitary lesion with enhancement. Contrast-enhanced CT revealed solitary lesions with enhancement within the pancreatic head, spleen, and liver. The patient underwent metastasectomies from the pancreas, spleen, and liver. The patient was discharged on postoperative day 22 without recurrence for 18 months after metastasectomy. Twelve years after primary resection and 2 years after metastasectomy, the patient died as a consequence of multiple metastases.

**Conclusions:**

We have presented a rare case of pancreatic and spleen metastases from ASPS. Resection by radical metastasectomy was successful without morbidity. Thus, for improved survival of patients with multiple metastases from ASPS, metastasectomy may be indicated. If multiple metastases are resectable, surgical approaches may be the preferred treatment.

## Background

In 1952, alveolar soft part sarcoma (ASPS) was reported for the first time by Christopherson, Foote, and Stwart [1]. ASPS is a rare tumor, which accounts for 0.5–1% of soft tissue sarcomas [2]. ASPS has been characterized as growing more slowly than other types of sarcoma, with a peak incidence around 30 years of age [2]. Occurrence sites are mostly in the extremities, thighs (41%), pelvis/iliac fossa (10%), and upper limbs (9%) [3]. Metastasis occurs in about 15–30% of cases, mainly at sites in the lungs, bones, and lymph nodes [3]. Metastases located elsewhere are rare, and to our knowledge, cases of ASPS with pancreatic and splenic metastases have thus far not been reported. Here, we present a case of ASPS that was successfully treated by resection of pancreatic and splenic metastases performed as elective procedures.

## Case presentation

**Fig. 1:**
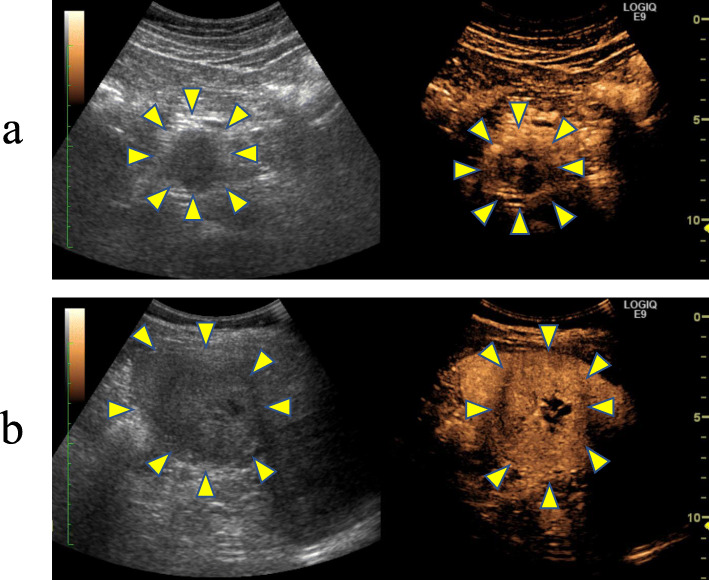
Abdominal ultrasonography (US) with and without contrast. Abdominal US with and without contrast revealed the presence of isodense masses of 34 mm (**a**) in the pancreatic head and 60 mm and in the spleen (**b**) (arrows)

**Fig. 2:**
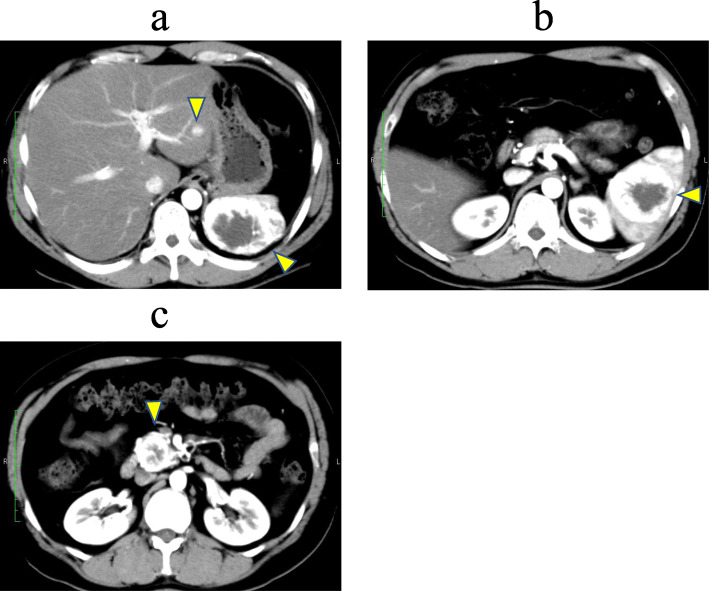
Contrast-enhanced computed tomography (CT) (horizontal slice). Contrast-enhanced CT revealed solitary lesions with enhancement in the liver (**a**), spleen (**b**), and pancreatic head (**c**) (arrow)

**Fig. 3:**
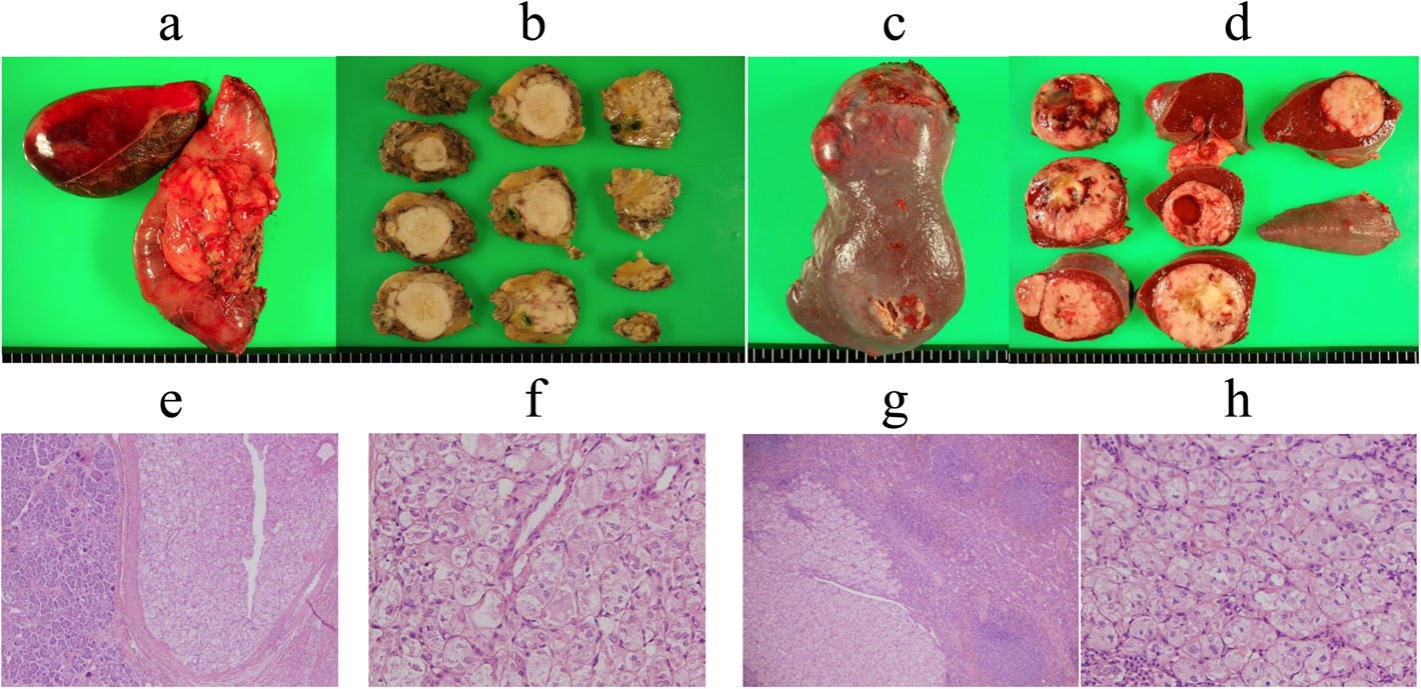
Findings of the resected specimen. The macroscopic and microscopic findings of the cut specimen revealed gray and clear borders between the tumor and pancreas (**a**, **b**) and spleen (**c**, **d**), and yellow and unclear border between the tumor and liver. Pathological examination showed that atypical cells with eosinophilic granular cytoplasm had proliferated to form alveolar solid nests in the pancreas (**e**, **f**) and spleen (**g**, **h**). The pathological examination further showed focal nodular hyperplasia at the liver

**Fig. 4:**
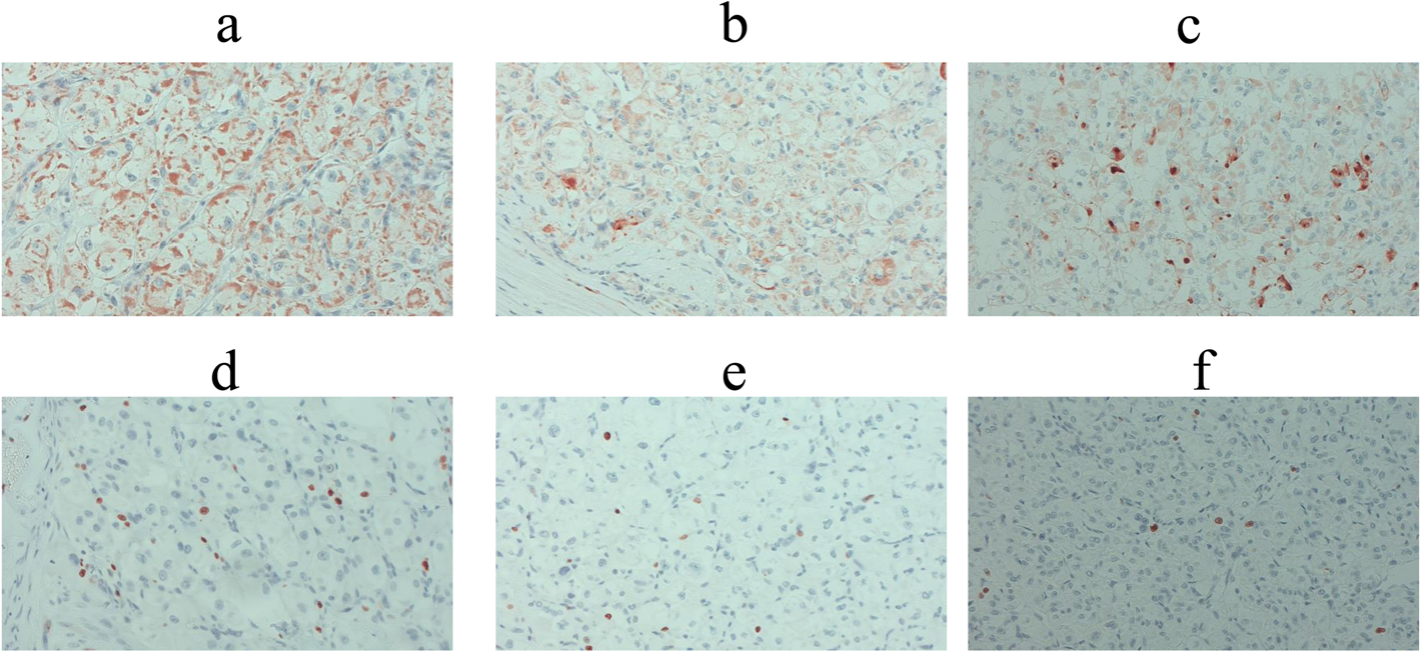
Immunohistochemistry analysis in primary ASPS, and pancreatic and splenic metastases (Desmin and Ki-67 original magnification x 200). Primary ASPS (**a**), pancreatic metastases (**b**), and splenic metastases (**c**) had Desmin-positive foci. The antigen Ki-67 proliferation index was < 10% in primary ASPS (**d**), pancreatic metastases (**e**), and splenic metastases (**f**)

A 41-year-old man who had undergone surgical resection of an ASPS of the anal region 10 years earlier presented to our hospital in 2012 because of a tumor of the pancreatic head detected via computed tomography (CT) during follow-up. In 2009, during follow-up, CT indicated lung metastasis. The patient underwent surgical resection of the lung, and in 2011, resection of the head skin was related to the metastasis. The abdominal ultrasonography (US) revealed the presence of isodense masses of 34 mm in the pancreatic head and 60 mm in the spleen. US with contrast revealed solitary lesions with enhancement (Fig. 1a, b). Contrast-enhanced CT revealed solitary lesions with enhancement located in the pancreatic head, spleen, and liver (Fig. 2a–c). Magnetic resonance cholangiopancreatography (MRCP) showed no stenosis of the pancreatic duct. Blood examinations revealed low hemoglobin (Hb) (12.3 g/dl), low hematocrit (Ht) (24.3%), and low total protein levels (6.6 g/dl). No further laboratory tests, including those for carcinoembryonic antigen (CEA) and carbohydrate antigen 19-9 (CA19-9), showed abnormal values. The patient was diagnosed with a neuroendocrine tumor or pancreatic metastasis of ASPS. Subtotal stomach-preserving pancreaticoduodenectomy (SSPPD) of the pancreatic head mass, resection of the spleen, and partial hepatectomy were performed. The operative time was 616 min, and the blood loss was approximately 1070 g. Gross examination revealed that the excision cut of the tumor was gray, and 40 mm in size, with a clear border between the tumor and pancreas. The excision margin of the spleen tumor was gray, 60 mm in size, and showed a clear border between the tumor and spleen. The excision cut of the liver tumor was yellow, 10 mm in size, and showed an unclear border between the tumor and liver. The pathological examination showed that atypical cells with eosinophilic cytoplasmic granules proliferated to form solid alveolar nests in both pancreas and spleen (Fig. 3a–h). Further examination showed focal nodular hyperplasia in the liver; immunohistochemistry analysis in primary ASPS and pancreatic and splenic metastases (Fig. 4a–f). Primary ASPS, pancreatic, and splenic metastases had Desmin-positive foci. The antigen Ki-67 proliferation index was < 10% in primary ASPS, pancreatic, and splenic metastases. The patient was discharged on postoperative day 22. However, the patient showed recurrence of multiple lung metastases at 18 months after metastasectomy. Twelve years after primary resection and 2 years after metastasectomy, the patient died as a consequence of multiple metastases.

## Conclusions

Overall, the patient survived primary resection of ASPS for 12 years, primary metastases for 8 years, and pancreatic and splenic metastases resection for 2 years. This noteworthy outcome of this case of metastatic ASPS was obtained by virtue of repeated operations on metastases that emerged over time.

A prior study of autopsied cases showed a prevalence of pancreatic metastases of 11.6% [4]. Pancreatic metastasis accounts for less than 2% of all pancreatic malignancies [5]. Specifically, the primary diseases of patients undergoing pancreatic metastasectomy were renal cell cancer in 61.7%, colon cancer in 7.8%, melanoma in 4.9%, and sarcoma in 4.9% of cases [5]. Pancreatic metastasis usually exhibits few symptoms and are often discovered by chance during follow-up examinations. However, their characterization is usually poorly defined, often reflecting the characteristic of the primary disease. Contrast-enhanced CT scans are required to distinguish the metastases from diseases such as pancreatic neuroendocrine tumors, acinar cell tumors, and others. It is necessary to confirm the clinical course, including the history and dynamics of the pancreatic hormones. EUS-FNAB is useful to establish a differential diagnosis, however should be used with caution because of the risk of bleeding, infection, pancreatitis, and dissemination. In this case, EUS-FNAB was not performed, because the clinical course of the patient suggested a metastatic lesion. At the time of metastatic pancreatic tumor detection, lymph node metastases have been reported in 33–38% of cases [6]. The metastatic forms of ASPS show hematogenous, lymphatic, and peritoneal dissemination. The frequency is often hematogenous metastasis, lung (63%), brain (19%), and bone (6%) [3]. Lymphatic metastasis is 2–7% [3, 7]. Common surgical procedures to treat pancreatic metastatic tumors include pancreaticoduodenectomy (PD), distal and middle pancreatectomy, total resection, and enucleation. However, the recurrence rate after atypical resection, such as enucleation, middle pancreatectomy, and duodenum-preserving pancreatic head resection, of pancreatic metastases from renal cell carcinoma is 50% [8]. It is generally assumed that a typical operation, such as PD and distal pancreatectomy, provides better treatment outcomes for pancreatic metastases [5]. The prognosis depends on the primary disease type. The average survival is about 4 months without surgical resection of ASPS, and the 5-year survival rate was about 20% [3]. Indications for resection of pancreatic metastases include their originating from primary renal cell carcinoma, a prolonged disease-free interval, and the absence of extra-pancreatic metastases [9]. We think, however, that surgical procedures could be selected whenever resection is possible and when the performance status of the patient was good after undergoing resection.

Pazopanib is an inhibitor of vascular endothelial growth factor (VEGF) receptor signaling [10]. In a study examining the efficacy of pazopanib for metastatic soft-tissue sarcoma (PALETTE) study in metastatic soft tissue sarcoma, the median progression-free survival (PFS) was 4.6 months, compared to 1.6 months in the placebo group [11]. In a phase 2 trial in patients with metastatic ASPS, the partial response rate following pazopanib was 16.7%, whereas the median PFS was 5.5 months [12]. Thus, because there no established chemotherapy that exists for these cases yet, surgical procedures are still the mainstay of treatment providing the best chance for long-term survival [13, 14]. Many metastases of ASPS occur in the lungs. The overall survival (OS) with resected lung metastases of ASPS amounts to 218 months, compared to about 63.5 months without resection [15]. However, it is not a statistical proof as it is a study of five patients resected and twelve patients unresected [15]. In 2015, median survival after the diagnosis of lung metastases was 34 months, and a 5-year survival rate was 64.1% for patients with lung metastases [16]. However, complete pulmonary metastasectomy has better survival than unresectable (3 years OS 32% vs. < 20%) [17]. Also, wide resection for local recurrence has better survival than unresectable (3 years OS 86% vs. 67%) [18]. In the literature in English, metastasectomies of the lung, brain, and local have shown favorable results with prolonged survival in selected patients [3, 14, 19–31] (Table 1). The pulmonary metastasectomy cases survived 60–132 months [3, 14, 19–22]. The local resection cases survived 6–300 months [14, 23, 24]. The brain metastasectomy cases survived 6–142 months [27–31]. Among the cases performed after metastasectomy, recurrence was found 2–240 months later. Long-term recurrence was 96 months at local [19] and 240 months at distant metastases [27]. The progression of ASPS is more slowly than other types of sarcoma [2]. Although pazopanib was not administered during the treatment of the present case, surgical procedures may be more effective if metastases can be removed. Here, we first report on the effect of metastasectomy of pancreatic and splenic metastases of ASPS.

**Table 1:** Characteristic of metastasectomy in case reports

author	Age	Sex	Primary site	Metastases at present	Metastases resection	Local therapy (Primary site)	RT	CT	Periods until recurrence (months)	Site of metastasis	Laterality	single / multiple	metastasectomy	RT	CT	Survival postmetastasectomy (month)	status
Baum ES [19]	14	M	Upper extremity	-	-	Exision	No	No	96	Lung	bilateral	multiple	Pulmonectomy	No	Yes	60	NED
Evans HL [20]	7	F	Scapula	-	-	Exision	Yes	No	40	Lung	bilateral	multiple	Pulmonectomy	No	Yes	69	NED
Kodama K [21]	23	F	Lower extremity	Lung	resection	Exision	No	Yes	-	Lung	bilateral	multiple	Pulmonectomy	No	No	98	NED
Portera CA [3]	-	-	unknown	Lung	resection	Exision	No	Yes	-	Lung	unknown	multiple	Pulmonectomy	No	Yes	132	NED
Sidi V [22]	11	M	Lower extremity	-	-	Exision	Yes	Yes	8	Lung	bilateral	multiple	Pulmonectomy	Yes	Yes	60	NED
van Ruth S [14]	22	F	Lower extremity	Lung	unknown	unknown	unknown	Yes	-	Lung	bilateral	multiple	Hepatectomy and nephrectomy	No	Yes	111	DOD
	18	F	Head and neck region (left temple)	-	-	Exision	Yes	No	36	Local	-	single	Exision	No	No	300	NED
Emmez H [23]	11	F	Head and neck region (left frontal lobe)	-	-	Exision	No	No	6	Local	-	single	Exision	Yes	Yes	6	NED
Wang Y [24]	9	F	Head and neck region (left eye)	-	-	Exision	Yes	No	84	Local	-	single	Exision	Yes	No	131	DOD
	12	F	Head and neck region (left eye)	-	-	Exision	No	No	2	Local	-	single	Exision	Yes	No	36	NED
	1	M	Head and neck region (left eye)	-	-	Exision	No	No	96	Local	-	single	Exision	Yes	No	-	NED
Wang M [25]	39	F	Lower extremity	-	-	Exision	No	No	120	Lung+brain	unilateral	single	Craniotomy	Yes	Yes	8	AWD
Daigeler A [26]	40	M	Lower extremity	-	-	Exision	No	No	9	Lung+brain	unknown	unknown	Exision	Yes	Yes	48	DOD
	21	M	Upper extremity	-	-	Exision	No	No	12	Lung+brain	unknown	unknown	Exision	Yes	Yes	79	DOD
	48	M	Lower extremity	-	-	Exision	No	No	7	Lung+brain+local	unknown	unknown	Exision	Yes	Yes	97	DOD
Ohashi H [27]	25	F	Buttock	-	-	Exision	No	No	240	Brain	unilateral	single	Craniotomy	No	No	142	DOD
Wronski M [28]	14	F	Lower extremity	Lung	resection	Exision	No	unknown	23	Brain	-	unknown	Craniotomy	No	No	73	DOD
	7	M	Head and neck region (tongue)	Lung	resection	Exision	No	unknown	23	Brain	-	unknown	Craniotomy	No	No	23	DOD
Ogose A [29]	61	M	Lower extremity	Lung, bone, bowel	unknown	Exision	No	unknown	84	Brain	-	unknown	Craniotomy	No	No	24	DOD
Tao X [30]	22	M	Upper extremity	Lung	unknown	Exision	No	Yes	-	Brain	unilateral	single	Craniotomy	No	No	69	AWD
	15	F	Chest	Lung	unknown	Exision	No	No	-	Brain	unilateral	single	Craniotomy	No	No	35	AWD
	26	F	Lower extremity	-	-	Exision	Yes	No	-	Brain	unilateral	single	Craniotomy	No	No	32	NED
	32	M	Lower extremity	Lung	unknown	Exision	Yes	No	-	Brain	unilateral	single	Craniotomy	No	No	31	NED
	25	M	Crus	Lung	unknown	Exision	No	No	-	Brain	unilateral	single	Craniotomy	No	No	20	DOD
	33	M	Lower extremity	Lung	unknown	Exision	No	No	-	Brain	unilateral	single	Craniotomy	No	No	25	AWD
	26	M	Lower extremity	-	-	Exision	No	No	-	Brain	unilateral	single	Craniotomy	No	No	14	NED
	23	M	Trunk/retroperitoneal/abdomen	-	-	Exision	No	No	-	Brain	unilateral	single	Craniotomy	No	No	6	NED
Kaushal-Deep SM [31]	20	M	Lower extremity	Brain	resection	Exision	No	No	-	Brain	bilateral	multiple	Craniotomy	Yes	Yes	12	AWD

*CT* chemotherapy, *RT* radiotherapy, *DOD* dead of disease, *NED* no evidence of disease, *AWD* alive with disease

In conclusion, we have presented a rare case of pancreatic and splenic metastases originating from ASPS. Radical metastasectomy by resection was performed successfully. Multiple metastases related to ASPS support the possibility that metastasectomy is associated with improved overall survival. If multiple metastases are found to be resectable, this procedure may be favorable candidates as part of surgical treatment.

## Data Availability

The patient data for this case report will not be shared to ensure patient confidentiality.

## Abbreviations

ASPS: Alveolar soft part sarcoma
CA19-9: Carbohydrate antigen 19-9
CEA: Carcinoembryonic antigen
CT: Computed tomography
EUS-FNAB: Endoscopic ultrasound fine-needle aspiration biopsy
MRCP: Magnetic resonance cholangiopancreatography
PD: Pancreaticoduodenectomy
PFS: Progression-free survival
OS: Overall survival
SSPPD: Subtotal stomach-preserving pancreaticoduodenectomy
US: Ultrasonography
VEGF: Vascular endothelial growth factor
